# Parent-therapist partnership to ELEVATE gross motor function in children with perinatal stroke: protocol for a mixed methods randomized controlled trial

**DOI:** 10.1186/s12887-022-03525-6

**Published:** 2022-08-10

**Authors:** Caitlin L. Hurd, Michelle Barnes, Christa M. Diot, Elizabeth G. Condliffe, Hana Alazem, Lesley Pritchard, Jennifer D. Zwicker, Anna McCormick, Man-Joe Watt, John Andersen, Adam Kirton, Jaynie F. Yang

**Affiliations:** 1 Department of Physical Therapy, Faculty of Rehabilitation Medicine, University of Alberta, 2-50 Corbett Hall, Edmonton, AB T6G 2G4 Canada; 2 Alberta Children’s Hospital, Calgary, AB Canada; 3 Departments of Pediatrics and Clinical Neurosciences, University of Calgary, Calgary, AB Canada; 4 Department of Pediatrics, University of Ottawa, and Children’s Hospital of Eastern Ontario, Ottawa, ON Canada; 5 School of Public Policy and Faculty of Kinesiology, University of Calgary, Calgary, AB Canada; 6 Department of Pediatrics, University of Alberta, and Glenrose Rehabilitation Hospital, Edmonton, AB Canada; 7 Department of Pediatrics and Department of Clinical Neurosciences, Alberta Children’s Hospital Research Institute, University of Calgary, Calgary, AB Canada

**Keywords:** Physical therapy, Early intervention, Cerebral palsy, Neurological rehabilitation, Qualitative

## Abstract

**Background:**

There is increasing evidence for early, active rehabilitation to enhance motor function following early brain injury. This is clear for interventions targeting the upper extremity, whereas passive treatment approaches for the lower extremity persist. The purpose of this trial is to evaluate the effectiveness of early, intensive rehabilitation targeting the lower extremity and delivered in a parent-therapist partnership model for children with perinatal stroke.

**Methods:**

We describe a protocol for a waitlist-control, single-blind, mixed methods effectiveness randomized controlled trial, with an embedded qualitative study using interpretative description. Participants are children with perinatal stroke aged eight months to three years with signs of hemiparesis. Participants will be randomly allocated to an immediate ELEVATE (Engaging the Lower Extremity Via Active Therapy Early) intervention group, or a waitlist-control group, who will receive usual care for six months. The ELEVATE intervention involves one hour of training four days per week for 12 weeks, with a pediatric therapist and a parent or guardian each delivering two sessions per week. The intervention targets the affected lower extremity by progressively challenging the child while standing and walking. The primary outcome measure is the Gross Motor Function Measure-66. Secondary outcomes include the Pediatric Quality of Life Inventory™, Young Children's Participation and Environment Measure, and an instrumented measure of spasticity. A cost-effectiveness analysis and qualitative component will explore benefit to costs ratios and parents’ perspectives of early, intensive rehabilitation, and their role as a partner in the rehabilitation, respectively.

**Discussion:**

This study has the potential to change current rehabilitation for young children with perinatal stroke if the ELEVATE intervention is effective. The parent interviews will provide further insight into benefits and challenges of a partnership model of rehabilitation. The mixed methods design will enable optimization for transfer of this collaborative approach into physical therapy practice.

**Trial registration:**

ClinicalTrials.gov NCT03672864. Registered 17 September 2018.

**Supplementary Information:**

The online version contains supplementary material available at 10.1186/s12887-022-03525-6.

## Background

Perinatal stroke occurs between 20 weeks gestation and 28 days postnatal life and is the leading cause of unilateral cerebral palsy [1] with an estimated incidence of 1 in 1600–3000 live births. Perinatal stroke may result in life-long motor impairments [1–4]. For example, the children typically have limitations with walking, which results in abnormal loading of the joints, premature musculoskeletal problems, reduced physical activity, and participation [5, 6]. Further, the sequelae from perinatal stroke can have a profound impact on family well-being [7].

Recent evidence indicates active interventions improve function [8], while passive interventions, such as bracing and botulinum toxin A injections, provide only short-term gains with unknown or suboptimal longer-term outcomes [9–11]. Early, intensive rehabilitation for the upper extremity has gained acceptance, but active rehabilitation for the lower extremity remains infrequent with little standardization between therapists and centres [12].

Our preliminary efficacy trial indicated that the ELEVATE (Engaging the Lower Extremity Via Active Therapy Early) intervention resulted in significantly greater improvements in gross motor function compared to a control group receiving usual care [13]. Children less than three years old with perinatal stroke tolerated one hour of intensive lower extremity activity, four days/week for twelve weeks.

Sufficient intensity of neurological rehabilitation is important [14], but it is often neither feasible nor cost-effective for physical therapists to administer high-intensity training for a prolonged period. Achieving this intensity is often better achieved by children’s parents or guardians, especially with very young children [15, 16]. Involvement of the family is essential for enhancing child outcomes [17], but there are limited reports of parents’ experiences with conducting home rehabilitation programs, especially as partners in therapy [18]. Furthermore, while family centered care advocates for parent-clinician partnerships, clinicians are cautioned against ‘downloading’ professionally driven interventions to families [19]. This study is a concurrent mixed methods design; the qualitative study will explore the parents’ perspectives of the intervention and their experiences as a partner in rehabilitation.

The purpose of this trial is to evaluate the effectiveness of early, intensive rehabilitation delivered using a parent-therapist partnership model for children with perinatal stroke. The trial was designed in collaboration with parents and therapists to build on our findings in the laboratory and determine if the intervention is effective in a real-world setting. We hypothesize that, as compared to a control group receiving usual care, young children with perinatal stroke and unilateral cerebral palsy (CP) who receive intensive lower extremity training delivered in a parent-therapist partnership will demonstrate greater improvement in gross motor function.

## Methods

### Experimental design

**Fig. 1:**
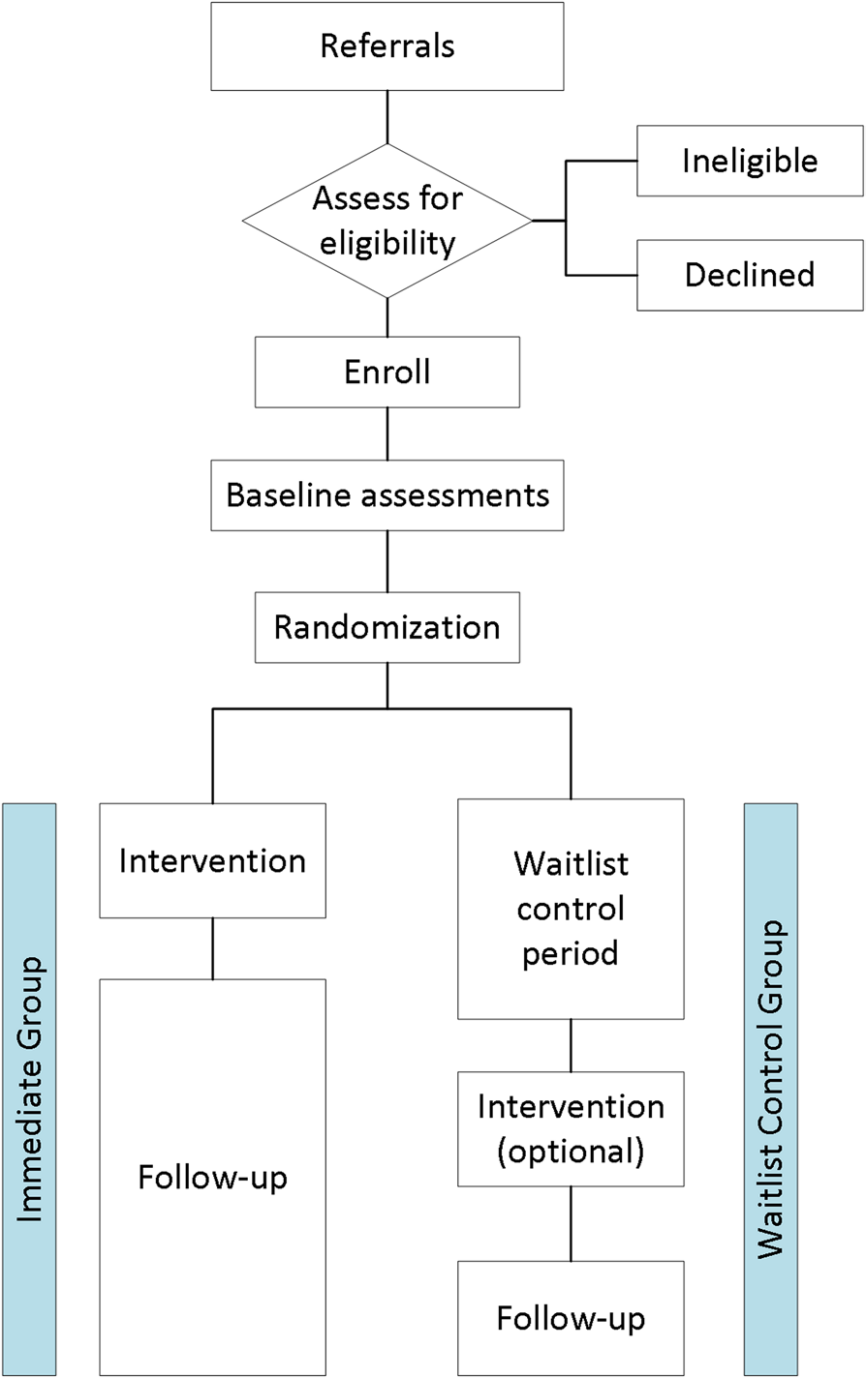
SPIRIT Flow Diagram. Participants are randomly allocated to either receive the ELEVATE intervention immediately (Immediate Group) or delay the intervention for 6 months (Waitlist-control Group) to serve as a usual care control

**Fig. 2:**
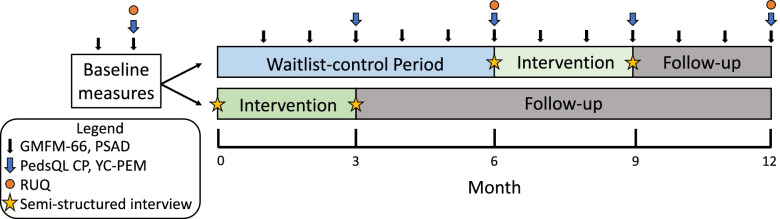
Experimental Design with Measurement Time Points. Participants are randomly allocated to either receive the ELEVATE intervention immediately (Immediate Group) or delay the intervention for 6 months (Waitlist-control Group) before they are offered the optional ELEVATE training. Children undergo monthly assessments for 12 months (including 9 months of follow-up for the Immediate Group and 3 months of follow-up for the Waitlist-control Group). Outcome measures are obtained as indicated by arrows and stars. GMFM-66: Gross Motor Function Measure-66; PSAD: Portable Spasticity Assessment Device; PedsQL CP: The Pediatric Quality of Life Inventory™ module for cerebral palsy; YC-PEM: Young Children's Participation and Environment Measure; RUQ: Resource Use Questionnaire

This is a three-centre (Edmonton [primary site], Calgary, and Ottawa), mixed methods study in which a qualitative study is embedded in a waitlist-control, single-blind randomized controlled trial (RCT). The children will be randomly allocated to either the Immediate Intervention Group (treatment) or the Waitlist-control Group (usual care) with follow-up (Fig. 1). The Immediate Intervention Group will undergo the three-month ELEVATE intervention immediately following randomization. The Waitlist-control Group will delay treatment for six months before initiating the ELEVATE intervention. The primary time point of interest is at six months in the study (Fig. 2). All children will undergo monthly assessments for 12 months in the study and will also be assessed at age four years of age to evaluate longer-term effects of training.

### Inclusion criteria

- children ages 8 months to 3 years
- medical history and physical exam consistent with perinatal stroke
- hemiparesis in the upper and/or lower extremity
- agreement from parent/guardian to adhere to the training and testing schedule

### Exclusion criteria

- bilateral motor impairment
- epileptic seizures that could interfere with training
- cognitive, behavioural or developmental impairments that preclude participation in the protocol
- botulinum toxin A injections or surgery in the lower extremities within the previous six months
- concurrent casting during the intervention phase (including constraint-induced movement therapy with casting)
- diagnosis associated with neurological/developmental regression
- parent unable to communicate (verbal and written) in English or French

### Randomization and stratification

Participants will undergo baseline assessments before randomization (Fig. 1). The randomization sequence was generated in Edmonton by a statistician. We used a permuted block design with a block size of 2–4. Randomization was stratified by city to ensure that the numbers allocated to the intervention and control groups will be roughly equal at each site. Their assignment is concealed until baseline assessments are complete, and are made available to the study sites through Research Electronic Data Capture (REDCap).

### Intervention

ELEVATE [13] will be administered one hour/day, four days/week, for twelve weeks. The ELEVATE intervention follows principles of motor learning and neuroplasticity [14], with high repetition of child-initiated activity. The activities will focus on weight-bearing on the feet with the aim to maximize recruitment of multiple, lower extremity muscle groups, especially on the affected side. Training may include activities such as kicking, squatting, navigating obstacles and walking on ramps and stairs. Children will train in soft-soled slippers, without braces or orthotics, to enhance active use of muscles throughout the lower extremity. Children who are not yet walking will be encouraged to walk with support while incorporating activities that increase weight bearing through the affected lower extremity and challenge their balance. As children begin to walk independently, their mobility will be further challenged by walking at different speeds, changing walking direction, and walking on unstable or more physically-demanding surfaces. The aim for intervention sessions is an hour of activity, which is anticipated to result in approximately 1000–3000 steps (as measured by step counters; Actigraph, FL), depending on the child’s age and ability [13]. Small ankle and foot weights (110 g and 20 g increments, respectively) will be used to increase work of the affected lower extremity, once children tolerate one hour of training [20]. Children with hemiparesis resulting from perinatal stroke typically walk asymmetrically, and adding ankle and foot weights to the affected lower extremity increases asymmetry of walking, which is necessary to induce learning [21]. Once children demonstrate more symmetrical gait with the weights, the amount of weight is increased to induce further learning [22].

### Parent therapist partnership

This effectiveness trial is designed to deliver the ELEVATE intervention by parents and therapists working in partnership. The partnership was considered an ideal model because it provides a high dose of intervention while supporting parents as partners in the rehabilitation process [23]. Physical therapists or physical therapy assistants working in public or private outpatient pediatric therapy centres will provide the intervention. The parent and therapist will each deliver two days of intervention per week. The parent will attend the intervention sessions with the therapist, thereby providing opportunities for observation and discussion. This will include reinforcement of the training principles, feedback on training and opportunities for progressions. Parents will be provided with a training manual describing suggested training activities [20].

### Intervention fidelity across centres

All treating therapists will be trained by the Principal Investigator and research physical therapist before the trial. Training will include background on activity-dependent neuroplasticity and an overview of the efficacy of early intensive rehabilitation. Practical training will include video examples and hands-on practice with young children. Treatment fidelity will be assessed throughout the trial using step-counts during intervention sessions, monthly video documentation, and biweekly meetings with staff at each site. Additional training sessions will be provided if training therapists indicate low confidence with the intervention.

## Outcomes

### Primary outcome measure

#### Gross motor function

The Gross Motor Function Measure-66 (GMFM-66) is the primary outcome measure, a criterion-referenced observational measure for assessing gross motor function in children with CP [24]. It has been validated for children > 0.5 years old and is a gold standard for measuring gross motor function in children with CP [25]. The assessing therapists will be pediatric physical therapists from the community trained by study personnel to administer the GMFM-66 and are masked to the child’s group assignment. All parents will be informed not to disclose the child’s group assignment to the assessing therapist. All assessments will be videotaped for reference. The GMFM-66 will be administered twice at baseline and monthly thereafter, for a total of 14 measurements per child. The two measures at baseline will be averaged to provide a more reliable estimate.

### Secondary outcome measures

#### Quality of life

Quality of life of the participants and their parents will be quantified at entry to the study, and again every 3 months using the Pediatric Quality of Life Inventory™, module for cerebral palsy (PedsQL CP) [26]. The PedsQL CP will be administered via online self-report through REDCap, or over the phone if respondents require assistance. The PedsQL is reliable and sensitive [26] and has been used extensively for this population. The children in this study are too young to self-report, so a parent proxy will be used. The scales in the CP module used are those recommended for children up to four years old [26]. The PedsQL also has a 36-item scale to measure the impact of a child’s health condition on the family, called Family Impact Module (PedsQL FIM). The scale has been widely used for parents of children with chronic health conditions, including perinatal stroke [7]. Three scores will result from this module, which reflect the parents’ health-related quality of life, family functioning and a total score.

#### Resource use and costing

Costs related to lower extremity therapy as well as resources used for treatment and management of the child’s cerebral palsy will be included over the study period, similar to a previously reported protocol [27]. The child will be the unit of analysis and utilization incurred by the participant, caregivers and/or the healthcare system will be assigned to the child for analysis. The Resource Use Questionnaire (RUQ) is a tool validated for use in participants with neurodevelopmental disabilities [28, 29] and will be used to collect information regarding resource use and out-of-pocket costs (e.g. physical or occupational therapy, materials and equipment, child-focussed recreation activities, etc.).

The RUQ will be administered at baseline, six months, the end of the one year study period and at the four-year-old follow up. The total cost per participant per month will be calculated and used in extrapolation. For the healthcare system perspective, costs will include physical or occupational therapy, child-focused recreation activities and additional services related to the child’s care paid for by the public healthcare system. For the societal perspective, costs will include all costs for the healthcare system perspective as well as parent lost productivity and parent out-of-pocket costs. For analysis using the societal perspective, a human capital approach [30] will be used to monetize lost caregiver productivity. Parent reports on the RUQ will include out-of-pocket costs for additional services, child-focused recreation activities, and materials and equipment. Lost productivity and out-of-pocket costs will be summed for the family of each participant to obtain total lost productivity and out of pocket costs for each participant over the study period.

The cost of the ELEVATE intervention will be estimated from the compensation to therapists including training, administration of the ELEVATE intervention and the cost of any materials and supplies associated with the intervention.

#### Participation

The Young Children's Participation and Environment Measure (YC-PEM) will be used to assess participation in three settings: home, daycare/preschool, and the community [31]. This questionnaire, completed by a parent or caregiver, was developed for children aged 0–5 years. The YC-PEM will be administered at baseline and every three months throughout the twelve-month study period and may be administered over the phone with research staff or via online self-report through REDCap.

#### Spasticity

We will use a newly developed device for measuring spasticity called the Portable Spasticity Assessment Device (PSAD, Nordic Neurostim, Denmark) [32, 33] to determine if the intervention reduces contracture or spasticity at the ankle, as seen in other active therapies for the lower extremities [32, 34]. Ankle joint range of motion, mechanical resistance caused by contracture and reflex-mediated spasticity can be measured using this device. We focus on the ankle, since this is the lower extremity joint very commonly affected by spasticity and is often the target of passive approaches including botulinum toxin, serial casting and orthoses [35]. Contracture, defined here as increased stiffness in joint movement cause by changes in intrinsic soft-tissue properties, will be reflected by the torque during slow stretch without any muscle activity. Spasticity will be indicated by the joint angle at which reflex EMG is generated in a fast stretch, similar to the angle of catch in the Tardieu Scale. This measure will be used at two study sites, because we only have two devices.

#### Reporting of co-interventions

During the six-month control period for the Waitlist-control Group and the follow-up period for both groups, parents will be asked to report any rehabilitation for their child’s lower extremity including the nature, duration and frequency. This will provide clarity on usual care, and the variations between sites.

### Sample size

The effect size on the primary outcome measure is estimated at 0.91, based on data from a cohort of children trained by their parents in our preliminary efficacy trial, as this is the more conservative estimate compared to the 1.67 effect size of children trained by a physical therapist. Twenty children per group are required to achieve statistical power of 0.8 and significance of 0.05. To account for cluster randomization with an estimated intra-cluster correlation coefficient of 0.02, a design effect of 1.4 is considered, which results in a total sample size of 56.

### Data management and analysis

Data will be entered in REDCap and analyzed at the primary study site. The primary time point of interest is at six months (Fig. 2), so change scores from baseline to six months will be compared between the ELEVATE intervention and control groups. Multiple linear regression models may be developed to assess the effect of variables (e.g. age at time of intervention) for each outcome. We will assess model fit and provide estimates with 95% confidence intervals.

### Cost-effectiveness analysis (CEA)

In the CEA, the ratio of the difference in mean cost between the ELEVATE and Waitlist-control groups to the difference in effectiveness scores (change in GMFM-66) between groups will be used to estimate an incremental cost-effectiveness ratio (ICER) from the publicly funded healthcare payer and societal perspectives. The mean effectiveness for each group will be compared using patient level regression. We will use Ordinary least squares (OLS) to assess the difference in effectiveness and the difference in cost between groups. We will control for a set of covariates including study site, age and baseline GMFM-66 scores. The mean cost per participant for each group will also be compared using participant-level regression. A bootstrapped analysis will be conducted for the CEA and results presented on a cost-effectiveness acceptable curve (CEAC).

### Trial monitoring

The trial is overseen by Health Canada because the PSAD is approved for investigational testing. The trial is monitored by the Quality Management in Clinical Research unit at the University of Alberta. The Research Ethics Board 3, University of Alberta, approved this study and the researchers are responsible for reporting protocol amendments and adverse events. Annual ethics renewals of the protocol are monitored by this Board.

## Qualitative study

### Design

Interpretative Description methodology [36] will be used as the methodological framework for the concurrent qualitative study to gain insight into the perspective of parents participating in a partnership with community therapists to administer the ELEVATE intervention. Interpretative Description has been chosen because this study intends to generate clinically relevant knowledge that is rich in description and interpretation and can be applied to improve clinical practice.

### Sample selection

Participants will be recruited from the multi-centre RCT and sampling will be purposive, to reflect the expected variation in experiences of participants [37]. We aim to interview parents with diverse backgrounds and experiences including individuals with varied socioeconomic status, multiple and single child families and single parent families. Sampling decisions will be made iteratively as information about participants’ experiences is explored and areas where further information is required becomes evident. Hence, a definitive sample size is not defined a priori but will be evaluated on an ongoing basis as data sufficiency becomes apparent or adequate to provide deep insight into the research question [38, 39]. We estimate an approximate sample size of 12–15 for the study, based on previous qualitative studies of parent experiences with home-based rehabilitation for children with CP [18].

### Data collection

Data will be collected through individual, semi-structured interviews at the start of intervention in the RCT and immediately following the intervention. Parents who choose not to participate in the RCT will be offered the opportunity to participate in a single interview to explore the barriers to participating in intensive rehabilitation. The interviews will follow a semi-structured interview guide (Supplementary File 1) at each time point. All interviews will be de-identified, audio recorded and transcribed verbatim.

### Data management and analysis

Thematic analysis will be used to organize and describe the data in rich detail. An inductive approach to thematic analysis will be used; themes will be strongly linked to the data [40], while interpreting findings with the intent to apply them to the practice of physical therapy. Data analysis will run concurrently with data collection to ensure that sampling and data collection are informed by emergent results. The coding process will involve interpretative thinking rather than simply recording and analyzing [41]. Field notes from interviews, including observations and interviewer impressions, will not be included in coding but will assist with interpreting transcripts.

### Rigour and credibility

The quality of this study will be enhanced by keeping a detailed record of activities, regular team meetings and ongoing reflexivity around the information being collected and the team members’ roles as clinician-researchers [42]. Detailed documentation will involve journaling and field notes following interviews. Modifications to the semi-structured interview guide will be documented as the team of researchers come to a deeper understanding of parents’ experiences throughout this study. Data analysis will be conducted collaboratively with at least two members of the research team.

## Discussion

Perinatal stroke results in life-long consequences for children and families. By intervening early, limited health care resources can be directed to children at an age when the effects are potentially largest. This study engages parents as partners so they become familiar with the principles of the approach. We anticipate that parent involvement will facilitate longer-term outcomes that will potentially enhance child motor development beyond the study period. We hypothesize that early improvements in gross motor function will enhance child outcomes and improve child and family quality of life. In addition, economic benefits may extend beyond our study period, for example, by delaying or avoiding the need for orthoses and botulinum toxin A injections, which we aim to capture at the four-year-old follow-up. Understanding parents’ experiences with the parent-therapist partnership model will allow for refinement of this approach to rehabilitation and more effective translation to clinical settings. Finally, partnering with key knowledge users, including families and pediatric clinicians, aims to bring about faster and more seamless adoption of the intervention if the results demonstrate effectiveness.

## Supplementary Information

**Additional file 1: Supplementary File 1.** Semi-structured interview guide. This document contains the semi-structured interview guide for interviews conducted with parents before and after the ELEVATE intervention.

## Data Availability

Not applicable. Results from this trial will be uploaded to clinicaltrials.gov upon completion.

## Abbreviations

ELEVATE: Engaging the Lower Extremity Via Active Therapy Early
CP: Cerebral Palsy
RCT: Randomized Controlled Trial
REDCap: Research Electronic Data Capture
GMFM-66: Gross Motor Function Measure-66
PedsQL CP: Pediatric Quality of Life Inventory™ module for cerebral palsy
YC-PEM: Young Children's Participation and Environment Measure
PSAD: Portable Spasticity Assessment Device
