# Supplementary material for: Parent-therapist partnership to ELEVATE gross motor function in children with perinatal stroke: protocol for a mixed methods randomized controlled trial
Source: BMC Pediatr. 2022 Aug 10;22:480. doi: 10.1186/s12887-022-03525-6 (PMC9364526; doi:10.1186/s12887-022-03525-6)
Supplement: Supplementary file 1 — Additional file 1: Supplementary File 1. Semi-structured interview guide. This document contains the semi-structured interview guide for interviews conducted with parents before and after the ELEVATE intervention. [file 12887_2022_3525_MOESM1_ESM.docx]

**Supplementary File 1**

Semi-structured interview guide

Interview 1 - Pre-training

1. Tell me about your family and your experiences with therapy for your child.
2. Why were you interested in participating in the study?
3. What are your expectations for the study? [probe how easy or difficult it will be]
4. How are you feeling about the upcoming training? [probe concerns, worries, hopes]
5. What are your goals related to this study? What would be the best outcome? Why?

Interview 2 - Post-training

1. Tell me about your experience with the study over the past three months.
2. What were your initial impressions of the training? Did they change over time?
3. Tell me about your experience being involved in delivering the training.
4. How did you incorporate the training into your life [probe who trained, when, etc.]
5. What do you wish you had known before starting the training?
6. How did you feel about being involved in your child’s training?
7. What would be helpful for therapists to know?
8. What are your thoughts about partnering with a therapist to provide the training to your child?
9. Follow-up on goals question from pre- intervention (remind parent of goals). Were goals reached, adjusted?
10. What would you say to a family of a child considering participating in the study?
11. What’s next for your child/family? Programs/services?
12. Is there anything else you’d like to mention about your experience?

Semi-structured interview guide – Single interview to explore barriers to participation

1. Tell me about your family and any experiences with rehabilitation so far.
2. What was interesting to you about the study? Why were you interested in getting more information from the research team?
3. What are the factors that you considered when making the decision to not enroll in the study?
4. Follow up on factors – e.g. how could they be changed to enable participation?
5. What would make a study or rehabilitation intervention interesting for you and your child to participate in?
   1. What expectations of you as a parent would make it possible to participate? (e.g. time commitment, role of parent, other possibilities)
6. Do you have any other general comments or experiences you’d like to share about accessing rehabilitation for your child?
